# Editorial: Doing more with less: neurosurgery strategies and tricks of the trade in the technological era

**DOI:** 10.3389/fsurg.2026.1993806

**Published:** 2026-10-26

**Authors:** Roberto Colasanti, Giovanni Raffa, Joham Choque-Velasquez, Bipin Chaurasia, Alessandro Di Rienzo

**Affiliations:** 1 Department of Neuroscience, Division of Neurosurgery, AUSL Romagna, Maurizio Bufalini Hospital, Cesena, Italy; 2 Division of Neurosurgery, BIOMORF Department, University of Messina, Messina, Italy; 3 Neurosurgery Unit, Hospital Regional Del Cusco, Cusco, Peru; 4 School of Medicine, Universidad Andina del Cusco, Cusco, Peru; 5 Department of Neurosurgery, Neurosurgery Clinic, College of Medical Sciences, Birgunj, Nepal; 6 Department of Neurosurgery, Azienda Ospedali Riuniti Ancona, Università Politecnica Delle Marche, Ancona, Italy

**Keywords:** classifications/grading systems, cutting-edge technolologies, doing more with less, low-cost solutions, minimally invasive approaches, neurosurgical planning, technical tricks, treatment algorithms

## Introduction

In recent decades, advancements in technology—such as operating microscopes, endoscopes, image guidance systems, intraoperative neuromonitoring (IONM), ultrasonic surgical aspirators, fluorescence-guided surgery, indocyanine green video angiography, machine learning-based radiomics, and risk prediction models—have significantly improved the accuracy, predictability, and safety of neurosurgical operations, ultimately enhancing patient outcomes (Wang et al.; Gu et al.) (1–4).

More recently, augmented reality tools and simulators have opened new avenues for preoperative surgical planning, while advancements in artificial intelligence and related technologies hold promise for optimizing early detection methodologies and refining treatment algorithms (1–3). However, a significant paradox remains: many of these technological innovations are still inaccessible in low- and middle-income countries (LMICs) (Doléagbénou et al., Zhang et al.). This has created an urgent clinical need for robust strategies that maximize surgical efficacy while minimizing resource consumption (5, 6).

The theme of this Special Issue, “Doing More with Less: Neurosurgery Strategies and Tricks of the Trade in the Technological Era”, addresses this challenge by exploring the integration of cutting-edge technologies to overcome neurosurgical hurdles, while emphasizing reliable, low-cost, and adaptive solutions suitable for resource-limited settings (Zhang et al.) (5–9). Furthermore, this issue highlights the development of novel predictive classifications, clinical algorithms, and decision-making systems aimed at optimizing resource allocation, preventing complications, and refining patient selection (Wang et al., Lv et al., Yuping et al.).

The “Doing more with less” philosophy finds numerous applications in neurosurgery, and this Editorial synthesizes the contributing articles into six cohesive, subspecialty-specific themes presented by the authors, ultimately aiming to bridge the gap between technological advancements and practical implementation in diverse healthcare settings.

### Minimally invasive and neuroendoscopic surgery

Minimally invasive neurosurgery significantly reduces surgical morbidity, accelerates postoperative recovery, and shortens hospital stays. In a comprehensive comparative study, Jalal et al. evaluated the clinical efficacy of neuroendoscopic surgery (NS) versus stereotactic aspiration (SA) for spontaneous intraparenchymal hemorrhage (Jalal et al.). Analyzing a cohort of 199 patients (97 NS, 102 SA), the investigators found that NS achieved a significantly higher median percentage reduction in hematoma volume compared to SA across all studied brain regions (92.90% vs. 22.20%, *p* < 0.001). This rapid clot evacuation translated into a more pronounced post-operative GCS improvement (4.0 vs. 0.5 points, *p* < 0.001). Most importantly, the direct visualization afforded by NS allowed for immediate intraoperative control of active bleeders, resulting in a reduced rate of symptomatic rebleeding (7.2% vs. 24.5%, *p* < 0.001) and a decrease in 30-day mortality (9.3% vs. 22.5%, *p* = 0.012) (Jalal et al.) (10–12).

Similarly, Liu et al. demonstrated the value of direct neuroendoscopic visualization in treating septated chronic subdural hematomas, where fibrous membranes impede conventional drainage (13, 14). Comparing 45 patients managed endoscopically with 52 managed via traditional burr hole drainage, they reported a dramatic reduction in postoperative recurrence in the endoscopic cohort (2.22% vs. 15.38%, *p* < 0.05) through systematic identification and fenestration of the fibrous septa (Liu et al.).

However, it is essential to recognize that a surgical technique is not *per se* minimally invasive merely due to the tools used (e.g., an endoscope) or the incision size; rather, it is truly minimally invasive only if it effectively minimizes the retraction and manipulation of neurovascular structures, thereby managing the condition while preventing post-operative deficits (15–18). In this context, Erginoglu et al. challenged the conventional belief that the endoscopic approach (EA) is inherently less traumatic than open microsurgical techniques. By comparing the interhemispheric transcallosal approach (ITA; *n* = 13) against EA (*n* = 10) for third ventricle colloid cysts, volumetric segmentation on early postoperative T2/FLAIR MRI images revealed that EA produced significantly larger volumes of parenchymal edema along the surgical tract compared to ITA (2,951.51 ± 2,083.87 mm^3^ vs. 349.45 ± 218.48 mm^3^, *p* < 0.001). Furthermore, late postoperative MRI demonstrated that gliosis, encephalomalacia, and structural parenchymal loss were entirely absent in the ITA group but occurred in 50% of EA cases (*p* = 0.007), suggesting that transcortical rigid endoscopic trajectories can lead to permanent parenchymal changes. ITA also achieved a significantly higher gross total resection rate than EA (92% vs. 50%, *p* = 0.039) (Erginoglu et al.).

For secondary cerebrospinal fluid (CSF) flow obstructions, selected cases may benefit from endoscopic techniques that offer elegant, shunt-free solutions, such as the endoscopic-assisted cisternostomy with Ommaya reservoir insertion for trapped temporal horn, effectively restoring physiological CSF pathways and avoiding the long-term complications associated with permanent ventriculoperitoneal shunting (AlSaiari et al.).

### Microsurgical and endovascular techniques for neurovascular diseases

The treatment of complex intracranial aneurysms necessitates continuous technical advancements (1, 3, 19).

For intricate posterior circulation pathologies, Mammadinova et al. detailed the “Stent-within-a-Stent” technique following telescopic flow diverter (FD) reconstruction for basilar artery aneurysms with pre-aneurysmal stenosis. This method stabilized the construct and effectively excluded the aneurysm, although the authors cautioned about the ischemic risks associated with multiple FDs in areas rich in perforators (Mammadinova et al.). Similarly, Zheng and Yu described the use of FD for complex mirror-like aneurysms located at the middle cerebral artery trifurcation, demonstrating that FD is a viable and effective alternative when traditional microsurgical clipping is not feasible.

In recent decades, significant advancements in endovascular techniques have transformed the management of neurovascular diseases, while microsurgical approaches continue to evolve and maintain specific indications. Consequently, vascular neurosurgeons must uphold their technical proficiency in addressing these challenging lesions (19–21).

Erginoglu et al. provided an intraoperative video analysis of the microsurgical clipping of a large, unruptured multilobular anterior communicating artery aneurysm associated with a vascular fenestration, emphasizing that fenestrations disrupt normal hemodynamic flow, thereby increasing the risk of aneurysm formation and surgical complexity. They highlighted essential technical strategies, including preemptive temporary clipping of the ipsilateral A1 segment to soften the aneurysm dome and high-magnification dissection to preserve the recurrent artery of Heubner and hypothalamic perforators (Erginoglu et al.).

In cases of catastrophic neurovascular complications, immediate salvage techniques are crucial. Li et al. presented an emergency repair method for an internal carotid artery rupture during a supratentorial craniotomy for a recurrent pituitary adenoma, successfully sealing the defect with an autologous crushed muscle patch secured via micro-suturing, achieving immediate hemostasis while ensuring long-term vessel patency (Li et al.).

Although cranial nerve syndromes are not classified as true neurovascular diseases, they arise from neurovascular conflicts where a compressing vessel affects the nerve, necessitating mastery of microsurgical techniques for effective and safe microvascular decompression.

In cases of recurrent cranial nerve syndromes, repeat surgery presents significant challenges that require adaptive microsurgical techniques. Liu et al. evaluated repeat posterior fossa exploration and external neurolysis in 38 patients with recurrent trigeminal neuralgia following microvascular decompression. While re-exploration revealed Teflon adhesion in 92.1% of cases, complete pain relief was achieved in 92% immediately and 86.8% long-term; however, aggressive neurolysis posed a risk of facial numbness. To mitigate this risk, the authors successfully employed a “limited neurolysis” strategy—isolating the Teflon and carefully dissecting adhesions without excessively stripping the nerve—demonstrating a critical technical approach for managing complex revision cases (Liu et al.).

The clinical significance of persistent embryonic pathways was comprehensively reviewed by Li and Yu, focusing on the persistent primitive hypoglossal artery, which often serves as the sole hemodynamically critical arterial supply to the posterior circulation, underscoring the importance of its preservation during surgical or endovascular interventions (Li et al.).

Finally, to ensure the safe and equitable delivery of neurovascular services, real-world registry studies are imperative. Liu et al. explored sex differences in 90-day clinical outcomes following mechanical thrombectomy in a prospective cohort of 334 Chinese patients with acute ischemic stroke. Their multivariate logistic regression analysis, adjusting for age, baseline stroke severity (NIHSS), and revascularization success (mTICI 2b-3), indicated that sex was not an independent predictor of 90-day favorable outcomes (adjusted OR 0.97, 95% CI 0.451–2.081, *p* = 0.939), reinforcing the equitable delivery of thrombectomy regardless of sex (Liu et al.).

### Digital health, smartphone-based research, and refined quality of care

A fundamental aspect of “Doing More with Less” is the strategic use of digital infrastructures to boost administrative efficiency, enhance clinical follow-up, and uphold baseline quality standards.

Turlip et al. introduced a methodological framework for smartphone-based physical activity research, employing smartphone accelerometers to continuously gather mobility metrics (daily steps, distance, and flights climbed), while eliminating the subjective biases associated with traditional patient-reported outcome measures (PROMs). By applying a 14-day moving average and normalizing datasets, they enabled machine learning models (e.g., Random Forest, achieving 86.7% accuracy) to predict individual recovery trajectories, demonstrating how consumer technology can transform objective longitudinal follow-up (Turlip et al.).

From an administrative standpoint, Haowei et al. assessed the digital empowerment of surgical privileging, implementing an informatics-driven, multidimensional refined privileging engine across 3,914 procedures (including 715 neurosurgeries), which resulted in a 44.3% reduction in review turnaround times (from 8.64 to 4.85 days, *p* < 0.001) (Haowei et al.).

In low-resource settings, the quality of care relies heavily on clinical discipline. Doléagbénou et al. evaluated postoperative care in the neurosurgery ward of the Sylvanus Olympio University Hospital in Lomé, Togo, uncovering significant procedural and organizational deficiencies, including inadequate medical documentation (adequate in only 52.9% of cases) and low checklist compliance, emphasizing that the key to minimizing morbidity in resource-limited regions lies in the rigorous implementation of standardized care protocols and regular audits, rather than immediate investiments in high-cost hardware (Doléagbénou et al.).

### Predictive modeling, radiomics, and biomarker-driven nomograms

Predictive models and nomograms are invaluable, cost-effective tools that assist clinicians in assessing complication risks and survival probabilities. For example, Lv et al. developed and validated a nomogram to predict one-year survival in patients with non-traumatic subarachnoid hemorrhage. By utilizing multivariate Cox regression on 825 patients from the MIMIC-IV database, the authors identified 11 independent risk factors, including age, respiratory rate, red blood cell count, platelets, electrolyte levels, and sepsis, achieving an AUC of 0.844 in the internal set and 0.807 in an external validation cohort of 290 patients from the Qinghai Provincial People's Hospital (Lv et al.).

Jiang et al. developed and validated a prognostic nomogram predicting 3-month functional recovery (mRS ≤ 2) in 289 surgical patients with spontaneous intracerebral hemorrhage. By integrating six imaging and clinical predictors—midline shift, hematoma volume, age, mean arterial pressure, BMI (revealing a protective “obesity paradox”), and GCS—the model demonstrated robust discriminative performance (AUC of 0.90 in training and 0.83 in validation), highlighting its utility as a low-cost, evidence-based tool for optimizing resource allocation and tailoring individual rehabilitation strategies (Jiang et al.).

Similarly, Wang et al. employed a machine learning approach to create a perioperative risk prediction model for pressure ulcers in craniotomy patients. After screening 700 patients using LASSO and backward stepwise regression, the final nomogram integrated eight clinically accessible predictors, including diabetes duration, BMI, albumin, urinary incontinence, prealbumin, age, hemoglobin, and skin temperature difference, demonstrating high predictive accuracy (AUC of 0.825 in training and 0.800 in validation) (Wang et al.).

In neuro-oncology, the preoperative prediction of molecular tumor characteristics can facilitate surgical planning without invasive biopsies, as demonstrated by Gu et al. (2026), who developed a radiomics nomogram to predict p53 protein overexpression in pituitary neuroendocrine tumors (PitNETs). By extracting radiomic features from preoperative contrast-enhanced MRI sequences in 186 patients and employing a two-step feature refinement process, the authors identified age, suprasellar invasion, and the radiomics score (Rad-score) as independent risk factors, successfully guiding personalized preoperative planning (Gu et al.).

Additionally, routine inflammatory and nutritional biomarkers can be synthesized to predict postoperative complications. Chang et al. (2025) explored the predictive value of combining the postoperative neutrophil-to-lymphocyte ratio (NLR) with the C-reactive protein-to-albumin (CRP/ALB) ratio for surgical site infections following spinal surgery. Their retrospective analysis of 380 patients revealed that both postoperative NLR (AUC = 0.791) and the postoperative CRP/ALB ratio (AUC = 0.878) are strong independent predictors of infection, underscoring how standard laboratory assays can enhance risk-adjusted patient surveillance (Chang et al.).

### Intraoperative neuromonitoring and neurosonography

Ensuring patient safety during complex spinal and cranial operations requires precise intraoperative physiological feedback. Severe spinal deformity surgeries carry significant risks of spinal cord injury. To reduce false-positive monitoring alerts, Gao et al. evaluated the adjunctive benefit of monitoring MEP recovery and EMG activity during the wake-up phase in 111 high-risk patients experiencing intraoperative MEP alerts. The authors found that tracking electrophysiological recovery during arousal offers vital complementary data to the traditional, physically demanding wake-up test, thereby lowering false-positive rates and enhancing risk assessment (Gao et al.).

In peripheral nerve surgery, the integration of technology improves functional preservation. Xing et al. explored the clinical utility of combining IONM with high-definition endoscopy during brachial plexus schwannoma resections. In a cohort of 20 patients, they achieved complete intracapsular enucleation with no permanent postoperative motor deficits, demonstrating that real-time electrical feedback, combined with close-up visualization, ensures meticulous nerve preservation (Xing et al.).

This focus on electrophysiological safety was echoed in a complex functional craniotomy reported by Tan et al., where surgeons attained a complete surgical cure of intractable post-traumatic epilepsy caused by a retained intracranial foreign body (a conical bamboo stick penetrating the temporal base). By employing real-time cortical electroencephalography monitoring, the surgeons accurately mapped the epileptogenic zone, enabling safe, targeted cortical resection (Tan et al.).

Intraoperative imaging has traditionally relied on high-cost, high-footprint intraoperative MRI; however, portable, real-time intraoperative ultrasonography (iUS) offers a cost-effective, high-utility alternative. Jamjoom et al. addressed the primary limitation of iUS—operator dependency and inter-observer variability in tumor boundary interpretation. By analyzing B-mode iUS tumor segmentations using the Dice Similarity Coefficient (DSC), the authors highlighted significant discrepancies, emphasizing the need to develop machine learning-augmented, convolutional neural network (CNN) models to standardize real-time boundary interpretation (Jamjoom et al.).

In low-resource settings, the clinical and economic advantages of portable iUS are particularly pronounced. Zhang et al. conducted a prospective, randomized controlled trial in Botswana, evaluating intraoperative color Doppler ultrasonography during the surgical evacuation of acute supratentorial intracranial hematomas. Comparing 34 patients managed with iUS navigation to 23 managed via conventional craniotomies, the authors reported a significantly higher rate of complete hematoma evacuation (>90% clearance) and a marked reduction in operative time, demonstrating that portable, low-cost iUS is a highly feasible and economically viable navigational alternative in resource-constrained regions (Zhang et al.).

Finally, neurosonology offers elegant, non-invasive physiological monitoring methods, as Çöven et al. reviewed the clinical utility of measuring the optic nerve sheath diameter (ONSD) via bedside transocular ultrasonography as a real-time surrogate for intracranial pressure (ICP) during high-volume epidural irrigation in epiduroscopy, effectively preventing secondary visual or perfusion complications (Çöven et al.).

### Enhanced recovery, dural sealing, and complex clinical traumas

Enhanced recovery after surgery (ERAS) protocols aim to accelerate recovery and reduce hospital stays. Kaewborisutsakul et al. (2025) conducted a double-blind RCT to evaluate whether adding ginger extract to a preoperative carbohydrate drink could enhance outcomes in elective neuro-oncologic craniotomies (Kaewborisutsakul et al.). In a cohort of 48 patients, those who consumed the ginger-enriched drink experienced a significant reduction in postoperative vomiting episodes (*p* < 0.05), demonstrating that standardized ginger extract is a safe, inexpensive, and globally accessible adjunct to neurosurgical ERAS protocols (Kaewborisutsakul et al.).

Achieving watertight dural closure is essential for preventing postoperative complications. Rossmann et al. established consensus recommendations for the clinical use of the PEG-coated collagen patch Hemopatch® based on a survey of 10 expert neurosurgeons, porcine workshops, and roundtable discussions. The panel outlined specific application techniques, recommending a 1 cm patch overlap, and showed that a new room-temperature configuration with a 36-month extended shelf life (“Hemopatch RT”) demonstrated superior tissue adherence, even in high-flow, wet areas like venous sinus bleeding (Rossmann et al.).

Wang et al. evaluated the biomechanical and infectious risks of autologous versus synthetic dural closure materials specifically for U-shaped dural incisions in a retrospective cohort of 172 patients, comparing standard continuous suturing (Method 1) with pericranium-assisted duraplasty (Method 2), which is used when intraoperative dural shrinkage hampers tension-free approximation. While both methods achieved excellent rates of watertight sealing, pericranium-assisted duraplasty posed a significantly higher risk of site-specific infections, particularly in the infratentorial compartment (12.82% overall infratentorial infection rate, *p* = 0.039). The authors emphasized that when utilizing autologous fascia, surgeons must carefully balance sealing efficacy against the increased local infection risk, especially in the posterior fossa (Wang et al.).

This Special Issue also addresses the management of rare and severe traumatic emergencies, including a systematic review and case report by Liu et al. on multiple thoracic vertebral compression fractures resulting from accidental low-voltage electric shock injury; they found that 75% of fractures were localized to the thoracic spine, where paraspinal muscle tetanic spasms impose extreme biomechanical stress, highlighting the importance of vigilance in diagnosing potential spinal fractures in individuals experiencing back pain after such injuries (Liu et al.).

Furthermore, Yang et al. showcased clinical adaptability in a complex transorbital penetrating injury caused by a long bamboo stick that penetrated the orbit and extended into the nasopharynx, successfully extracting the organic foreign body through a minimally invasive, endoscopic endonasal approach, thereby avoiding significant collateral damage and ensuring excellent visual recovery (Yang et al.).

## Conclusion and future directions

The articles compiled within this Special Issue illustrate the multifaceted concept of “Doing More with Less” in today's technological landscape. Neurosurgery must balance the rapid adoption of advanced, high-cost technological systems with the practical development of flexible, accessible, and cost-effective solutions. This includes the implementation of portable neurosonography in LMICs (Zhang et al.), the creation of universally accessible nutritional protocols in ERAS (Kaewborisutsakul et al.), the development of robust, laboratory-derived prognostic nomograms (Lv et al., Yuping et al.), or the enhancements of surgical authorization software (Haowei et al.). These studies emphasize that surgical excellence is fundamentally rooted in “clinical creativity”, disciplined care, and meticulous technique. Looking ahead, the integration of artificial intelligence and digital health should prioritize bridging the global healthcare gap, ensuring that high-quality, safe, and precise neurosurgical care is accessible to patients across all socioeconomic backgrounds.
